# Supplementary material for: Parainfluenza Virus 5 Infection in Neurological Disease and Encephalitis of Cattle
Source: Int J Mol Sci. 2020 Jan 13;21(2):498. doi: 10.3390/ijms21020498 (PMC7013525; doi:10.3390/ijms21020498)
Supplement: Supplementary file 1 [file ijms-21-00498-s001.zip › FigureS1_ISH_IF_PIV5_NegCo.pdf]

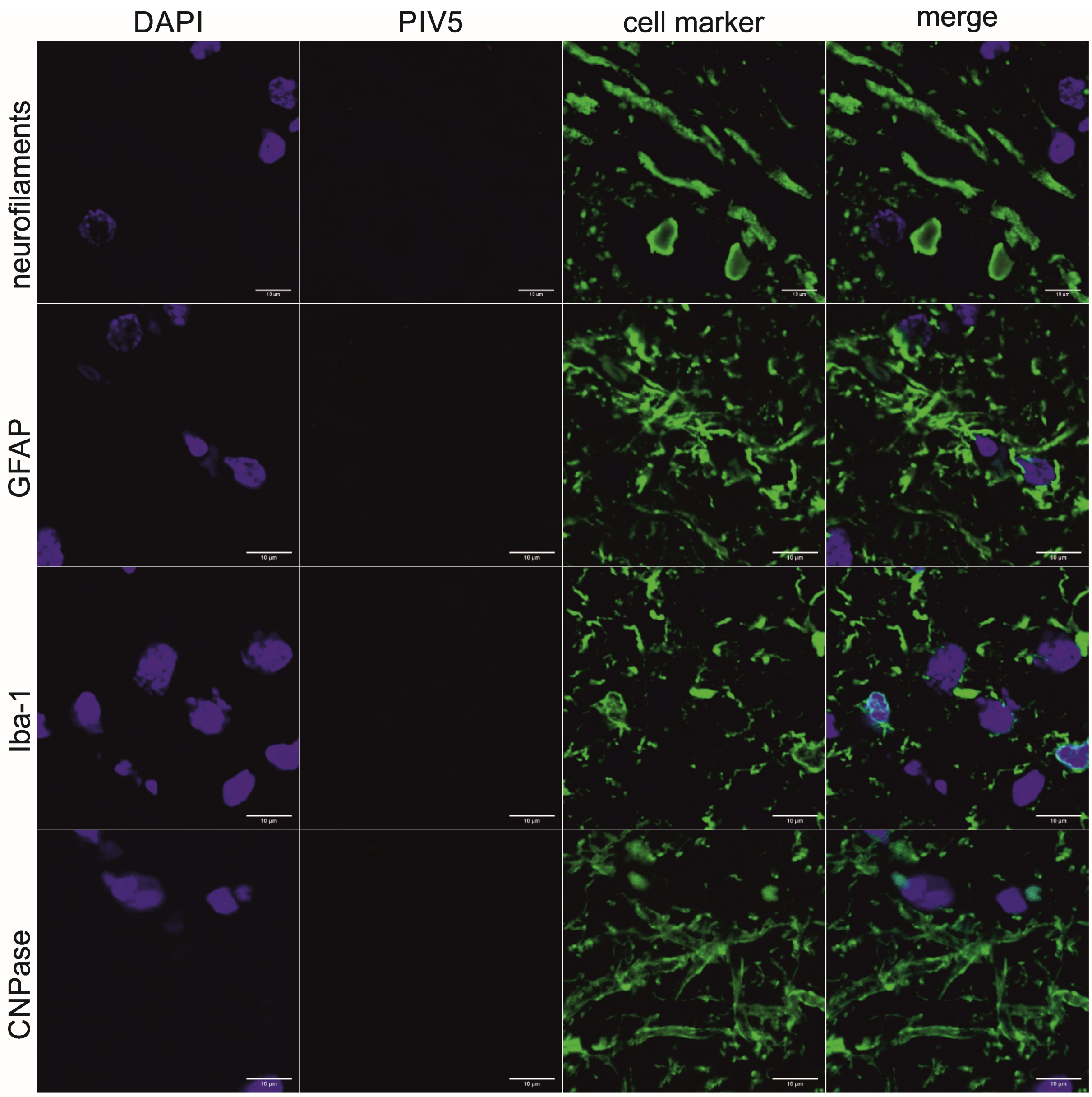

Figure S1: Negative controls of correlation of PIV5 RNA with different brain cell types. Combination of fluorescent *in situ* hybridization (ISH) using the PIV5 RNAscope probe with immunofluorescence (IF) using different cell markers of negative control animal 31292. Cell markers used are neurofilaments clone 2F11, glial fibrillary acidic protein (GFAP), ionized calcium-binding adapter molecule 1 (Iba-1), 2',3'-cyclic-nucleotide 3'-phosphodiesterase clone 11-5B (CNPase).Nuclei are stained in blue, PIV5 RNA is stained in red and different cell markers are stained in green. No PIV5 RNA can be detected in any of the stainings.
