# Supplementary material for: Parainfluenza Virus 5 Infection in Neurological Disease and Encephalitis of Cattle
Source: Int J Mol Sci. 2020 Jan 13;21(2):498. doi: 10.3390/ijms21020498 (PMC7013525; doi:10.3390/ijms21020498)
Supplement: Supplementary file 1 [file ijms-21-00498-s001.zip › FigureS2_standardcurves.pdf]

A

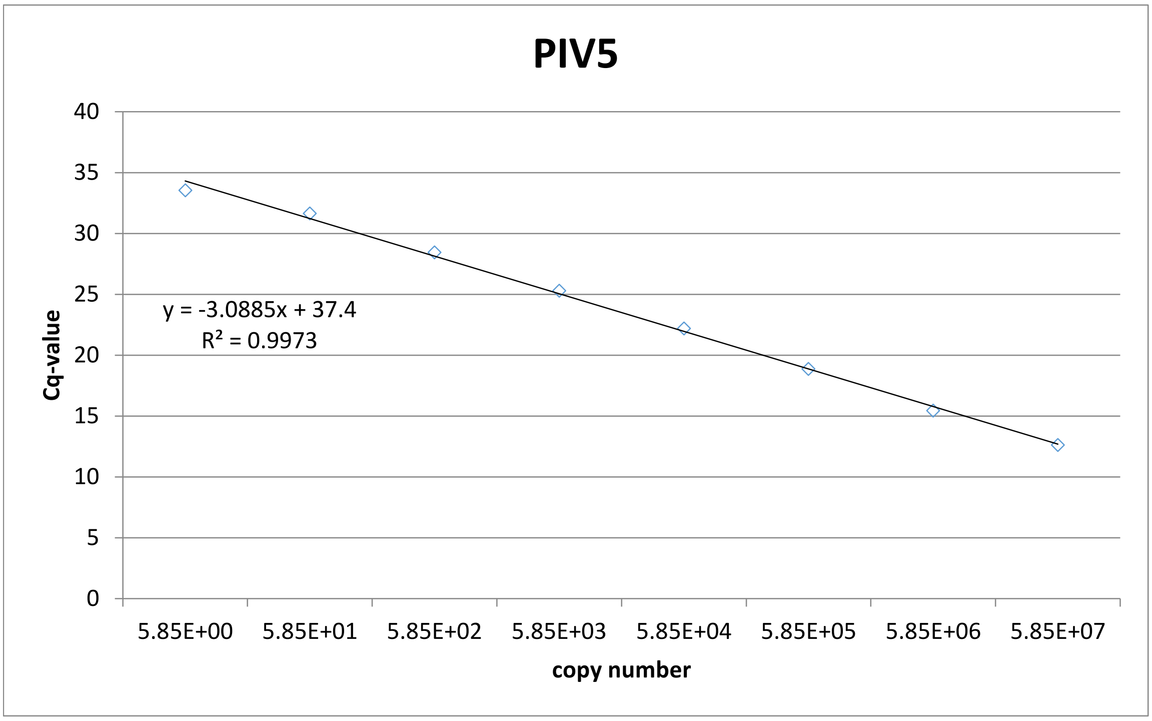

PIV5
Cq-value
y = -3.0885x + 37.4
R² = 0.9973
40
35
30
25
20
15
10
5
0
5.85E+00
5.85E+01
5.85E+02
5.85E+03
5.85E+04
5.85E+05
5.85E+06
5.85E+07
copy number

B

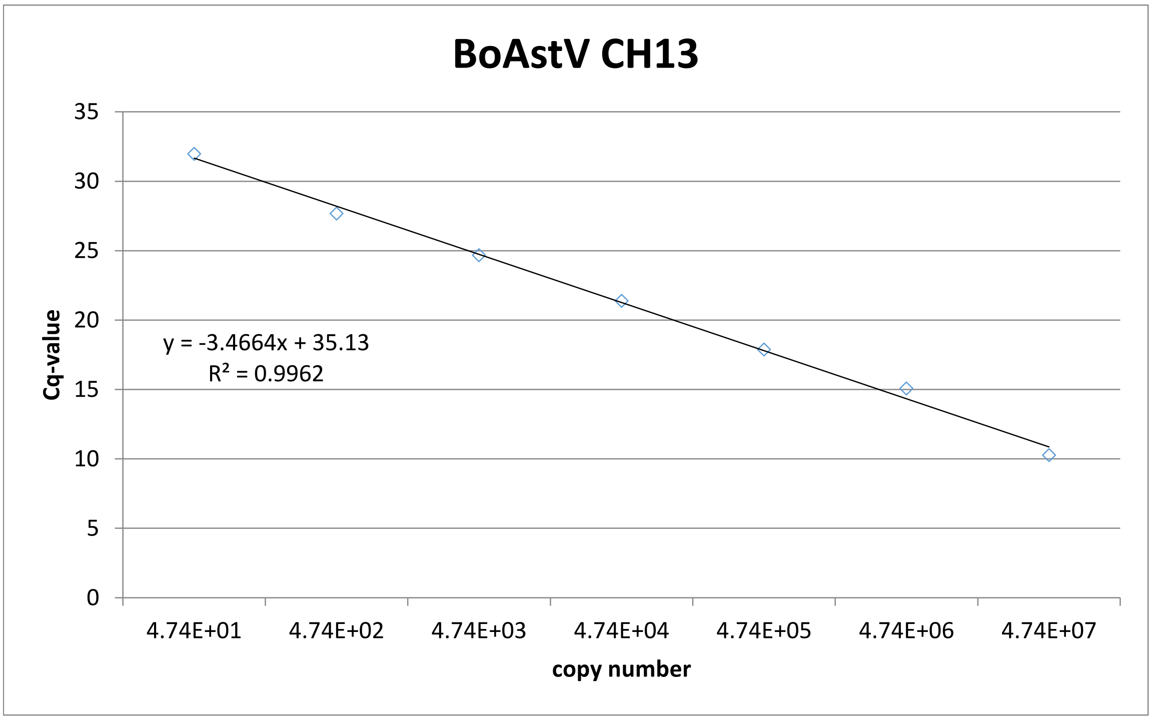

BoAstV CH13
Cq-value
y = -3.4664x + 35.13
R² = 0.9962
35
30
25
20
15
10
5
0
4.74E+01
4.74E+02
4.74E+03
4.74E+04
4.74E+05
4.74E+06
4.74E+07
copy number

C

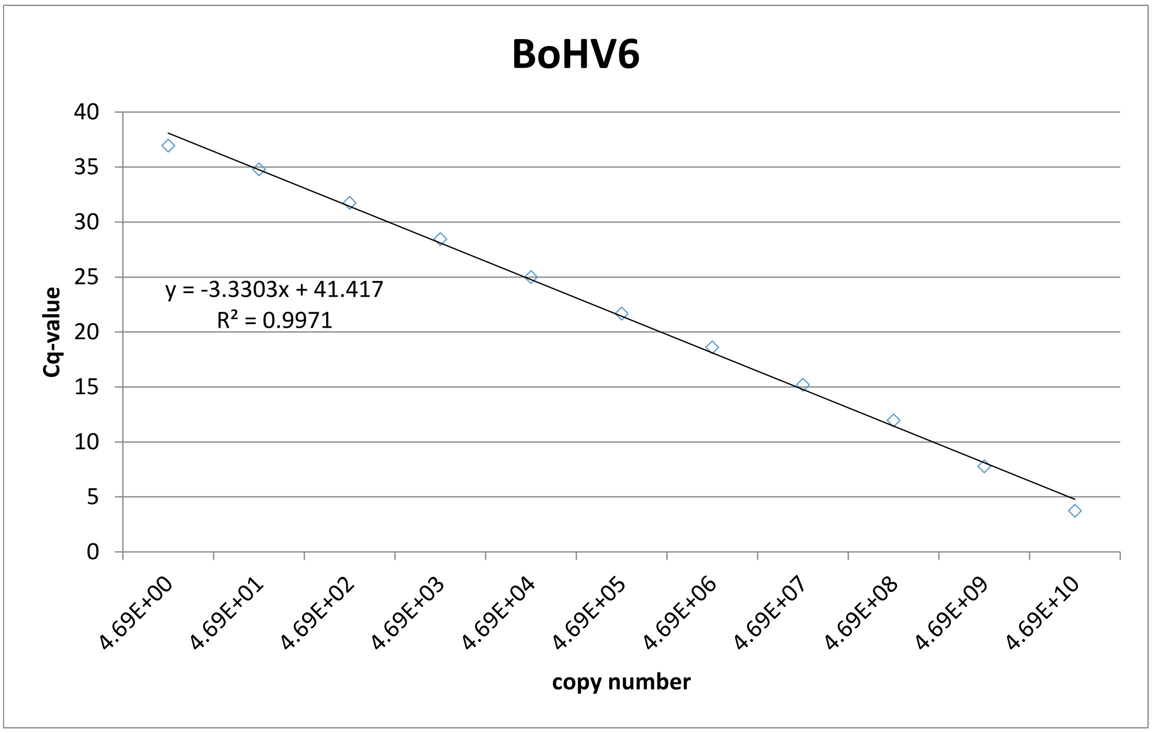

Figure S2: Standard curves of defined viral copy numbers in relation to Cq values. Cq values are plotted against 10-fold dilution series of defined copy numbers of PIV5 (A), BoAstV (B) and BoHV6 (C). For every standard curve a formula for copy number calculation from Cq values is given as well as the coefficient of determination.
